# Comparative analysis of mechanical conditions in bone union following first metatarsophalangeal joint arthrodesis with varied locking plate positions: A finite element analysis

**DOI:** 10.1371/journal.pone.0303752

**Published:** 2024-05-16

**Authors:** Agnieszka Sabik, Karol Daszkiewicz, Wojciech Witkowski, Piotr Łuczkiewicz

**Affiliations:** 1 Department of Mechanics of Materials and Structures, Faculty of Civil and Environmental Engineering Gdańsk University of Technology, Narutowicza Gdańsk, Poland; 2 II Clinic of Orthopaedics and Kinetic Organ Traumatology, Medical University of Gdansk, Smoluchowskiego, Gdańsk, Poland; Khalifa University of Science and Technology, UNITED ARAB EMIRATES

## Abstract

**Background:**

First metatarsophalangeal joint arthrodesis is a typical medical treatment performed in cases of arthritis or joint deformity. The gold standard for this procedure is arthrodesis stabilisation with the dorsally positioned plate. However, according to the authors’ previous studies, medially positioned plate provides greater bending stiffness. It is worth to compare the mechanical conditions for bone formation in the fracture callus for both placements of the locking plate.

**Methods:**

Two finite element models of the first metatarsophalangeal joint with the dorsally and medially positioned plate were defined in the Abaqus software to simulate differentiation of the fracture callus. A simplified load application, i.e. one single step per each day and the diffusion of the mesenchymal stem cells into the fracture region were assumed in an iterative hardening process. The changes of the mesenchymal stem cells into different phenotypes during the callus stiffening were governed by the octahedral shear strain and interstitial fluid velocity according to Prendergast mechanoregulation theory. Basing on the obtained results the progress of the cartilage and bone tissues formation and their distribution within the callus were compared between two models.

**Findings:**

The obtained results suggest that after 6 weeks of simulation the healing progress is in general comparable for both plates. However, earlier closing of external callus was observed for the medially positioned plate which had greater vertical bending stiffness. This process enables faster internal callus hardening and promotes symmetrical bridging.

## Introduction

First metatarsophalangeal joint (MTP-1) arthrodesis is described as safe procedure with revision rate ranges from 0 to 24% [1–3]. A good outcome following this procedure depends on stability and strength of the MTP-1 joint fusion during the postoperative period that should allow achieving bone union. For this reason, the current investigation focuses on new techniques of arthrodesis fixation strong enough to allow early weight bearing [4–8]. In biomechanical studies authors state that a dorsal plate is a gold standard for fixation of first MTP-1 arthrodesis [1, 9, 10]. On the other hand, Kuik and Łuczkiewicz [11] in medical trials suggest that medially positioned locking plate is valuable alternative to the dorsal plate. A follow-up experimental and finite element analysis [12] provided mechanical explanation of differences between both plate positions. However, in the literature there is a lack of studies assessing the influence of MTP-1 fixation construct on the biomechanical stimulus provided at the fracture site.

Bone regeneration is a complex biological process, which is essential for the formation of a callus that bridges the end of the bones and allows bone union. This process is carried out by specific key cells including mesenchymal stem cells (MSC), osteoblasts and osteoclasts. Given a sufficient blood supply, the course of bone healing seems to be mainly influenced by mechanical factors including interstitial fluid pressure, tissue tension and fluid flow. These stimuli are sensed by transmembrane proteins and convert into biomechanical reaction and synthesis of a variety of osteogenic markers [13, 14]. In 1979 Perren proposed a theory for the regulation of mesenchymal cell pool differentiation by assuming, that this process is determined by interfragmentary strain [15]. However, in further investigation it was demonstrated, that mechanoregulation of bone healing is governed not only by strain but also by the interstitial fluid velocity within the callus tissue [16, 17]. In these theories low mechanical stimulation favours osteoblast differentiation from the mesenchymal cell pool, intermediate stimulation favours chondrocyte differentiation and high level of stimulation favours fibroblast differentiation. The best way to obtain strain field and fluid velocity distribution is application of finite element method [18, 19], because they are very difficult to be measured in vivo.

The amount and distribution of specific tissues in callus during differentiation process may be calculated in numerical simulations to predict the healing outcome for different stabilization constructs [20]. Taking the above into account, in the present work the bone healing process is modelled basing on theory of Lacroix and Prendergast [21], which assumes the octahedral shear strain (*γ*) and interstitial fluid velocity (*v*) as biomechanical factors stimulating the tissue differentiation [17]. The model is still being developed and widely used to simulate healing of long bones [22–24] and other bone tissues, like surrounding of the dental implants [25–27] or spine vertebrae [28]. Usually, an iterative healing progress with a simplified load subjection, i.e. one single step per each day [21] is simulated.

The study aims to compare numerically conditions responsible for healing process in MTP-1 joint arthrodesis with dorsally or medially positioned locking plate. For this reason two finite element models of the MTP-1 joint are created which differ in the placement of the locking plate with respect to the load direction. The fracture gap is surrounded by the soft callus whose transformation into the bony tissue is simulated in an iterative process assuming diffusion of the MSC into the callus and simplified load subjection, i.e. one load cycle per each day [29]. As the load case the toe-off phase of the gait cycle is assumed. The average maximum load value depends on the postoperative treatment. The results obtained with the use of two models enable comparison of the progress of the cartilage and bone tissues formation and their distribution within the callus during the its hardening.

## Methods

### Bone healing model

The tissues in the chosen bone healing model, as proposed by Prendergast, due to the high water content, must be considered as biphasic materials [17, 29]. For this purpose the porous material theory, e.g., implemented in Abaqus software, is successfully used [30, 31]. Lacroix and Prendergast [21] argued that the fracture callus is initially filled with the granulation tissue. The mesenchymal stem cells entering the callus, depending on the value of the stimulus index *S* [25, 29], differentiate into fibrous connective tissue (3 < *S*), cartilage (1 < *S* < 3), immature bone (0.267 < *S* < 1) and mature bone (0 < *S* < 0.267). The value of *S* is governed by the octahedral shear strain (*γ*) and interstitial fluid velocity (*v*) [17]:

$$S=\frac{\gamma }{a}+\frac{v}{b},$$ (1)

where *a* = 0.0375 and *b* = 3μm/s [17]. According to [32], the excessive fluid flow is identified if S > 6. In such a case in the present study, the respective tissue is assumed to be initial granulation tissue. The differentiation process is modelled iteratively, assuming that each iteration refers approximately to 1 day of healing [21], but this time should not be over-interpreted [22].

In the course of healing the MSC infiltrate the callus. The rate of change of cells concentration is simulated as a diffusion phenomenon [21, 22, 25]:

$$\frac{\partial c}{\partial t}=D\nabla^{2}c$$ (2)

where *D* is the diffusion coefficient and *c* is a normalized cell concentration [33] *c*∈ 〈0,1〉.

Each day the callus is subjected to load corresponding to the chosen phase of the gait cycle. Since the tissues are considered as porous, the transient soil consolidation problem is resolved with the effective stress theory which assumes that the total stress **σ**_*tot*_ acting on point is partly carried by the solid phase, **σ**_*eff*_, and the wetting liquid (*p*>0), depending on the saturation (*s*):

$$\sigma_{eff}=\sigma_{tot}+spI$$ (3)

**Fig 1:**
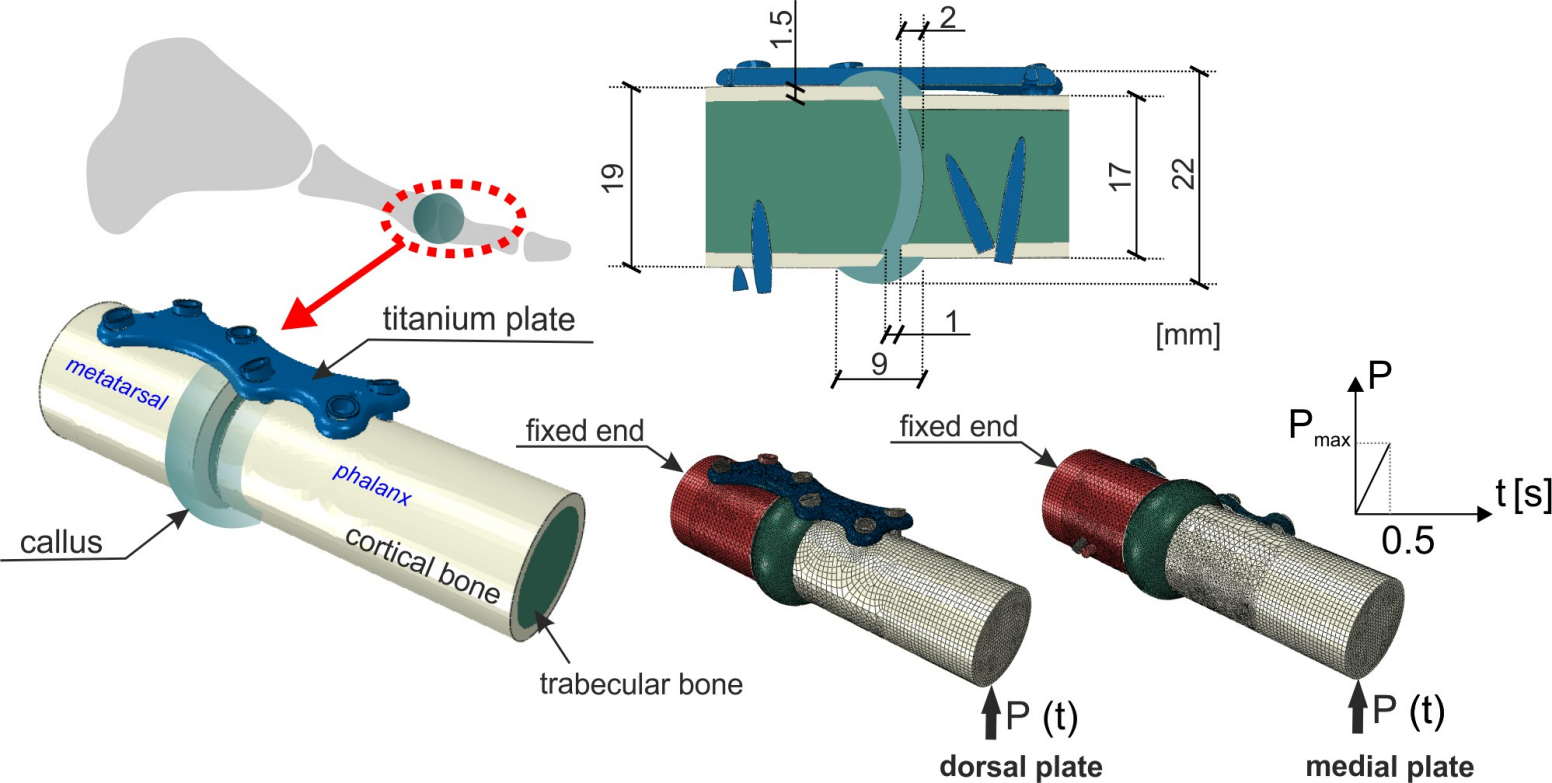
Geometry of the model and loading conditions.

The biomechanical stimulus (1) is calculated at the peak load (*P* = *P*_max_, see Fig 1). According to the identified tissue phenotype and taking into account a smoothing procedure, new material properties *M*_*i*_ of callus are recalculated. In each (*i+*1)^th^ day new property is assumed as an 10-day average value, similarly as in [22, 34]:

$$M_{i+1}=\frac{1}{10}\sum_{k=i‐9}^{i}M_{k}$$ (4)

where *M*_*k*_ stands for a material property (Young modulus, permeability, etc.) in *k*^th^ day. The final effective material property value of the considered region is established taking into account the local concentration (*c*) of the MSC:

$$M_{i+1}^{eff}=c\cdot M_{i+1}+(1-c)\cdot M_{gran}$$ (5)

where *M*_*gran*_ indicates the property of initial granulation tissue. Newly established material properties are used as input data in the subsequent iteration.

### Numerical model

The analysis is performed with the use of Abaqus 2021.HF12 software together with additional user subroutine USDFLD and Python scripts that provide the linking of the results between successive iterations.

In the present study the geometry of the MTP-1 joint model is simplified. The metatarsal and phalanx bones’ shape are modelled as cylindrical. During arthrodesis in the standard fixation technique [35], the bones of the MTP-1 joint are brought closer to each other. However, due to the possible lack of congruency of the MTP-1 joint [36], an average small gap of 1 mm is assumed [32, 37] between the metatarsal’s and phalanx’s cortical fracture bone fragments. Small callus inclusion (0.5 mm) into the trabecular bone is taken into account [21, 22, 29]. These and other dimensions together with loading conditions are given in Fig 1.

All materials except titanium (used for fixation plate and screw) are modelled as poroelastic and their properties are listed in the Table 1. If not specified, the data is taken after [21]. The elastic properties of titanium are *E* = 110 GPa, *v* = 0.37. Its plastic behaviour is described by the Johnson-Cook constitutive equation, see [12, 38].

**Table 1:** Properties of poroelastic materials.

	Cortical bone	Trabecular bone	Granulation tissue	Fibrous connective tissue	Cartilage	Immature bone	Mature bone
*E*	20000	700 ^a)^	1	2	10	1000	6000
*v*	0.3	0.3 ^a)^	0.17	0.17	0.17	0.3	0.3
*k*	10^−17^	9.81·10^−8^ ^b)^	10^−14^	10^−14^	5·10^−15^	10^−13^	3.7·10^−13^
*n*	0.04 ^c)^	0.8 ^b)^	0.8 ^c)^	0.8 ^c)^	0.8 ^c)^	0.8 ^c)^	0.8 ^c)^

*E*–Young modulus [MPa], *v*–Poisson ratio [–], *k*–permeability [m^4^/N·s], *n–*porosity

a) [39]

b) [40, 41]

c) [29]

**Fig 2:**
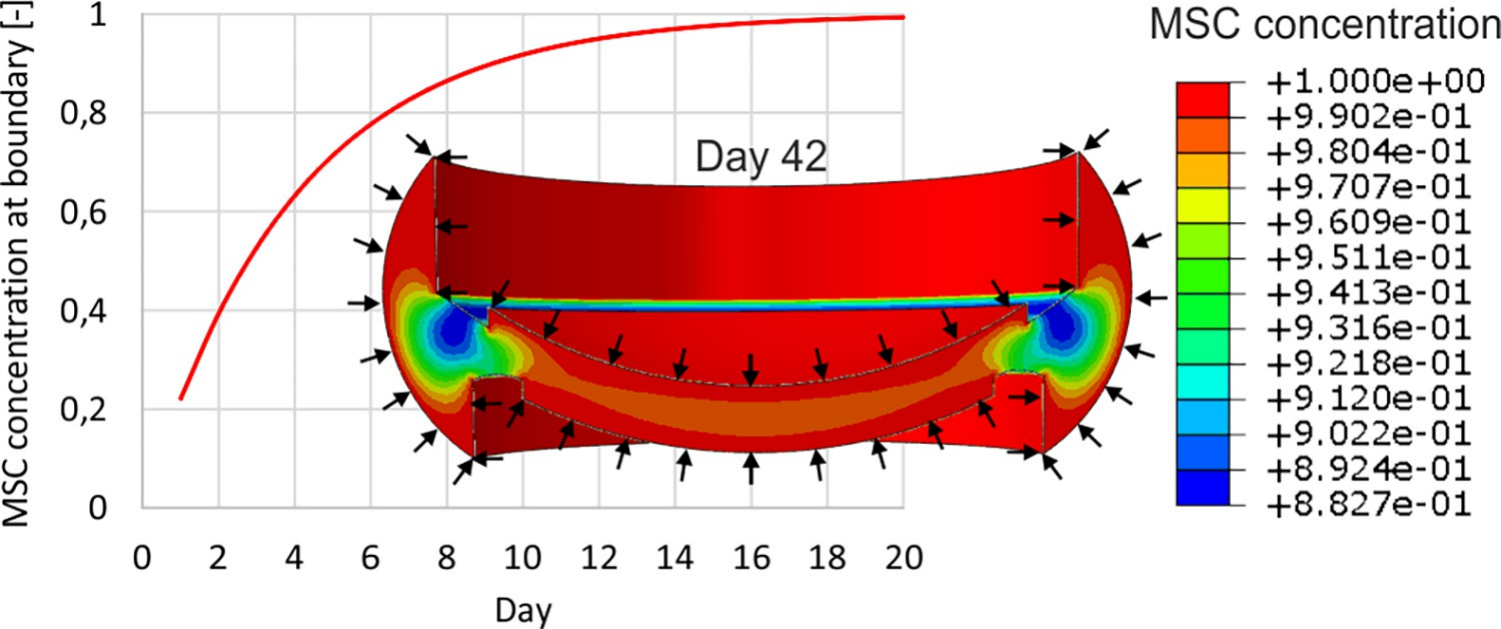
Conditions for MSC transfer; exemplary MSC concentration distribution in 42^th^ day.

The transport of MSC entering the fracture callus is described as diffusion. When Abaqus is used, due to the compatibility of heat and mass transport equations, the heat transfer analysis is carried out. The callus is discretized with 52883 C3D8RPT elements. It is assumed that the mesenchymal stem cells origin from the surrounding tissues, periosteum and trabecular bone (Fig 2). In order to avoid too fast stiffening of the regions next to the callus borders, similarly as in [42], the concentration at the boundaries varies in time such that the maximum value is reached after ca. 3 weeks *c*_*bound*_ = 1.0−*exp*(−*t*/4) [days], (see Fig 2). The value of the diffusion coefficient *D* (2) is estimated such that it provides steady state cell concentration after 16 weeks of healing [21]. The distribution of MSC is the initial condition in the subsequent mechanical stimulation (consolidation) analysis.

The tissues are meshed with reduced integrated pore-pressure elements, whereas the screws and fixation plate are modelled with standard 3D Stress elements. The entire model consists of 615905 finite elements.

At each day of healing one load cycle is realized. In the study the toe-off gait phase is chosen as a representative one, assuming 1 Hz load frequency. Fig 1 depicts the adopted mechanical boundary conditions. The stimulus (1) is calculated for the maximum load value in each finite element. Two loading histories are considered. The first corresponds to the postoperative treatment providing forefoot stress decrease [43]. In this case it is assumed that the maximum load applied to the phalanx (Fig 1) during first 4 weeks reaches P_max_ = 25 N, after that it increases linearly up to P_max_ = 50 N during 4 subsequent weeks. In the second loading history a constant peak load value P_max_ = 50N is adopted from the beginning, which is assumed to match the conditions without forefoot stress reduction.

The tie constraint is applied between the callus and the bones and between the bones and screws. Normal hard and tangential contact with friction coefficient equal to 1.0 are defined at the interfaces between the screws and the titanium plate. The external surfaces of the model parts are impermeable. Such an assumption is usually made [21, 22, 33, 44], according to the histological experiments which reveal that the callus tissues are covered by fascia [45].

Moreover, it has to be emphasized, that usually in studies employing similar finite element model (FEM) [33, 42], the poroelastic tissues are considered as fully saturated. However, in the present study a partial saturation is assumed to account for the capillary effects. Otherwise, in the case of the dorsal plate, large suction arising in the callus and trabecular bone due to loading, limits the movability of the phalanx in significant manner. According to [12], and assuming that the presence of the callus filled with granulation tissue should not influence the movability of the phalanx essentially, it is expected that due to 50N load the ratio of vertical translation of the phalanx between dorsal vs medial plate position approaches ca. 1.5. If the tissues are considered as fully saturated in the present poroelastic model, the qualitatively opposite relation is obtained, i.e. the dorsal plate provides much lower movability than the medially positioned plate. In this case the deformability of the callus is significantly restricted due to the large suction it undergoes. To find the remedy for this problem several analyses were performed with varying boundary conditions, like free drainage from the callus and/or bone-ends or variation of permeability with void ratio. Accounting for capillary effects was the only remedying technique that enables the higher deformability of the callus. Similar investigations are reported in [46]. Thus, finally the linear sorption in the material of the callus and trabecular bone was assumed. The saturation *s* = 1.0 was set for no suction and value *s* = 0.8 for maximum suction that was identified in the fully saturated case, i.e. *p* = −1.0 MPa. Then, assuming quasi-static loading conditions in the poroelastic model, the initial vertical translation of the phalanx under 50 N load is 1.5 mm for the dorsal plate and 0.89 mm for the medial plate, which correspond well to the ratio of 1.5 [12]. If 1Hz load is considered, then deflections 1.05 mm and 0.87 mm are obtained for the dorsal and medial plates, respectively.

## Results

**Fig 3:**
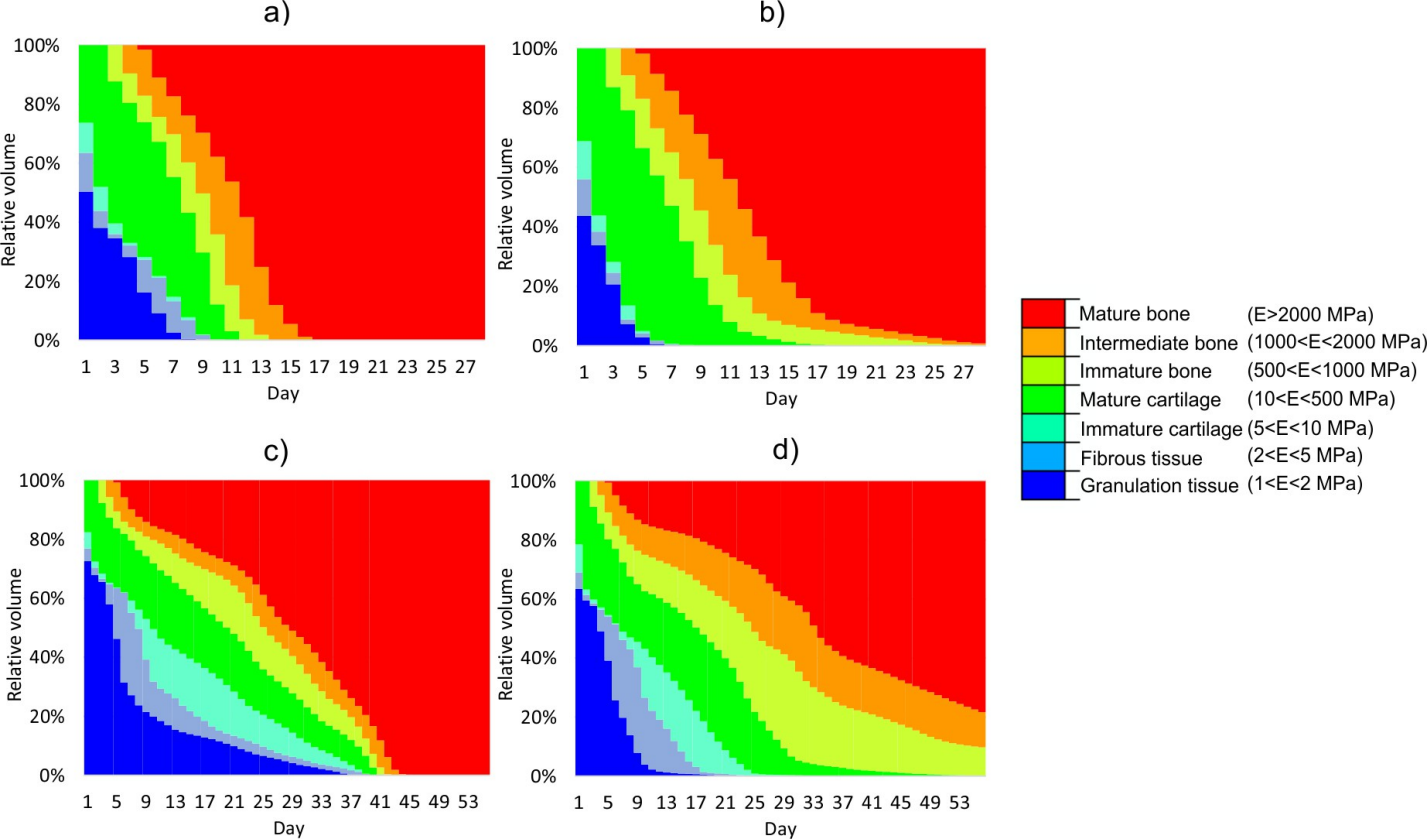
Relative volume of different tissue phenotypes in each day of healing: a) dorsal plate, P_max_ = 25N; b) medial plate, P_max_ = 25N; c) dorsal plate, P_max_ = 50N; d) medial plate, P_max_ = 50N.

Fig 3 shows the percentage (relative volumes) of the specific tissue phenotypes in the callus during each day of simulated healing depending on the plate position. It is assumed that each tissue phenotype is identified basing on the elastic modulus achieved in considered region of the callus [22, 33, 47]. This figure shows that the total callus transformation into the bone tissues is achieved during: 12 days (dorsal plate) and 18 days (medial plate) in the case of lower load level (25 N) and 41 days (dorsal plate) and 52 days (medial plate) for greater load level (50 N). These observations seem to expose the advantage of the dorsal plate over the medial one. However, it is reasonable to investigate the hardening process in more detail, not only the final outcome. Especially, the distribution of the cartilage and bone tissues within the callus is of the great importance.

**Fig 4:**
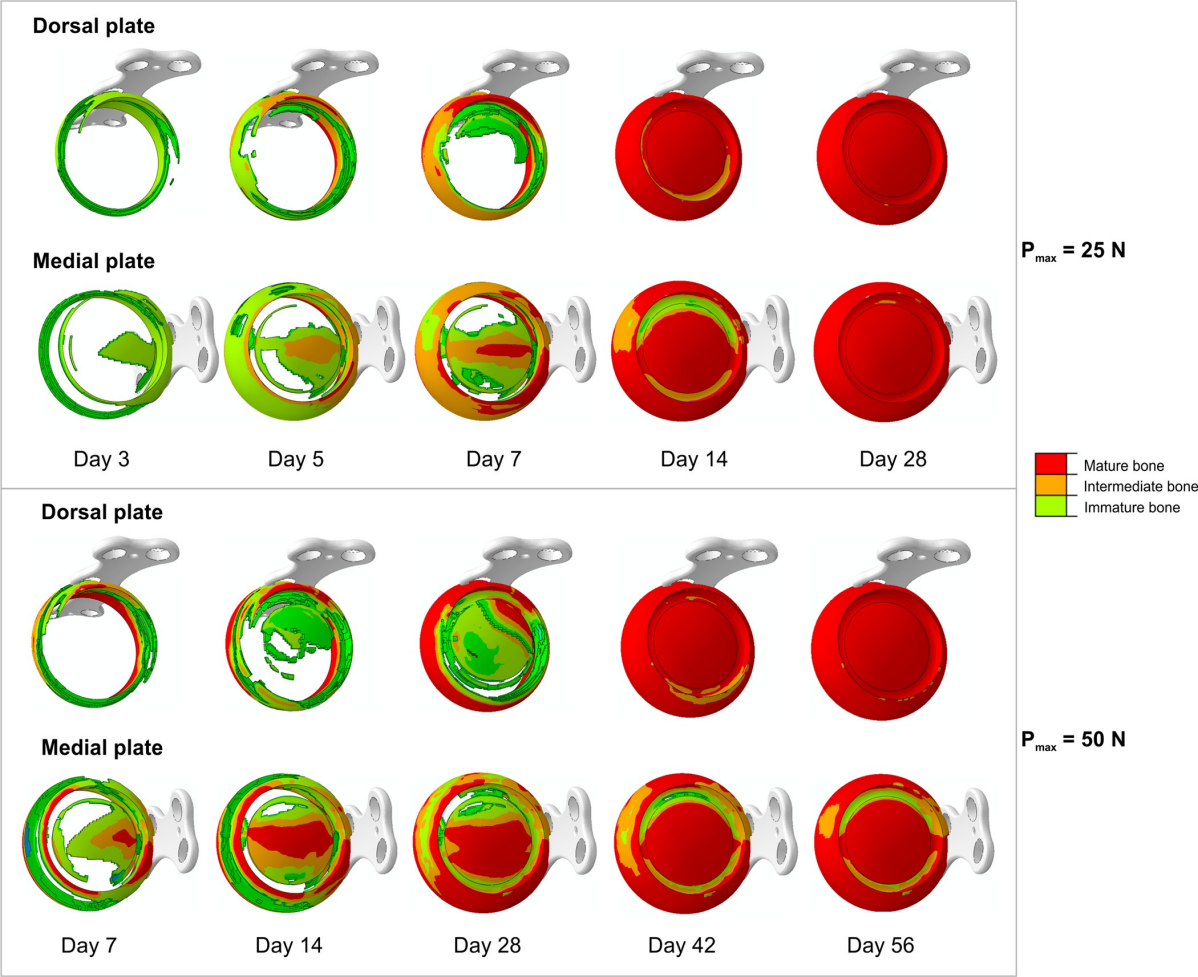
Progress of bone tissue differentiation during healing.

Thus, Fig 4 depicts the progression of bone tissues distribution in the callus. It reveals that the medial plate promotes faster bone formation within the gap. The bone tissues in the internal callus are observed already in the first week of healing, irrespectively of the level of load applied. Since the differences in results between models with different placement of locking plate are much more pronounced for larger load, only the load case of maximum 50 N is studied in detail.

**Fig 5:**
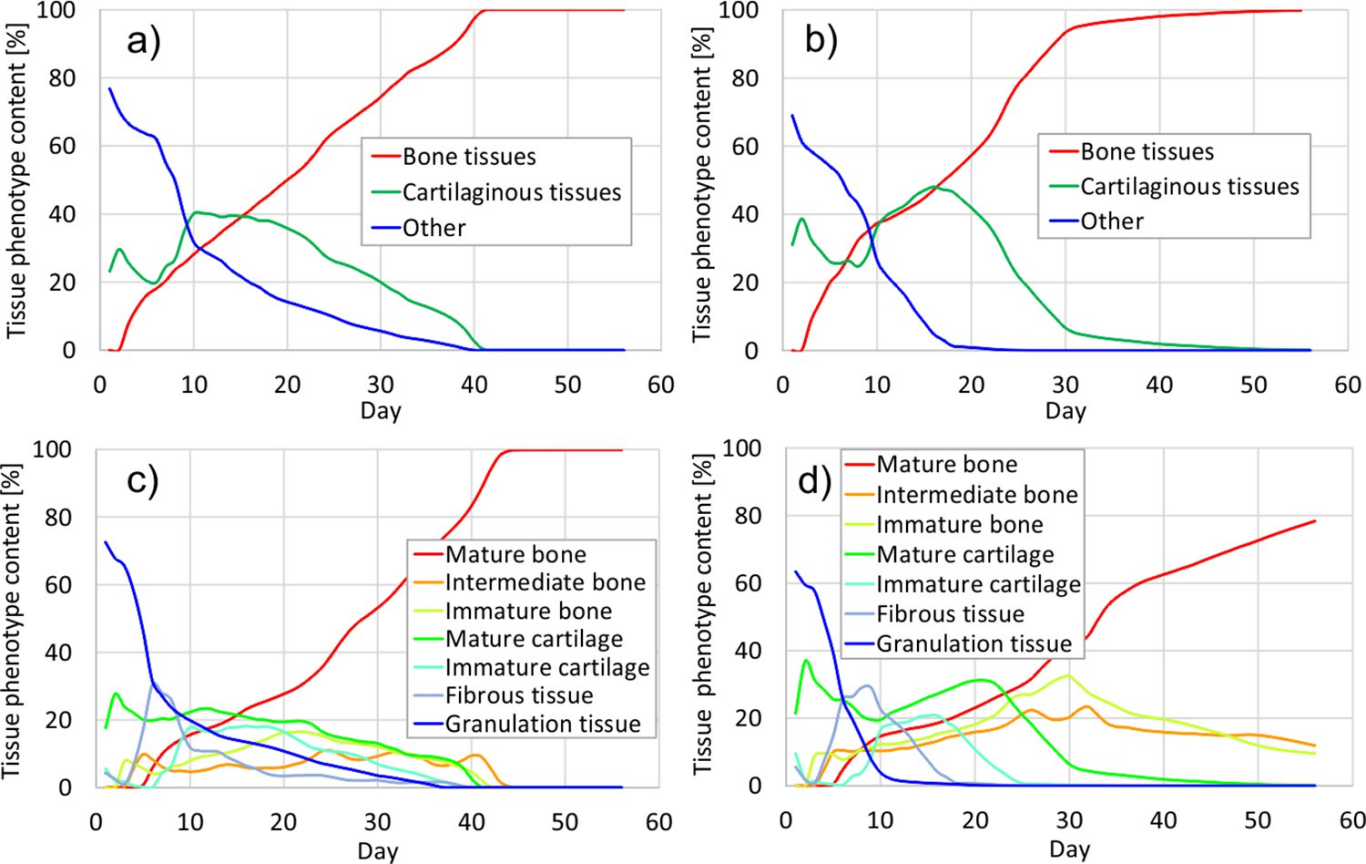
Changes of tissue types content during healing, P_max_ = 50 N; without specific phenotypes distinction: a) dorsal plate, b) medial plate; with specific phenotypes distinction: c) dorsal plate, d) medial plate.

For load equal to 50 N Fig 5 presents the changes of content of tissue types (bone, cartilaginous and other) during healing with and without the specific phenotype distinction. It is observed, that medially positioned plate provides earlier formation of the hard callus, i.e. at the end of the 3th week the callus is built of cartilaginous and bone tissues only, Fig 5B. In contrast, such outcome for dorsal plate is achieved after 6 weeks, Fig 5A. Nonetheless, at this moment the callus stabilized with the dorsal plate is composed totally of mature bone, Fig 5C, whereas in the fracture site assembled with medial plate some content (~20%) of intermediate and immature bone are present, see Fig 5D.

**Fig 6:**
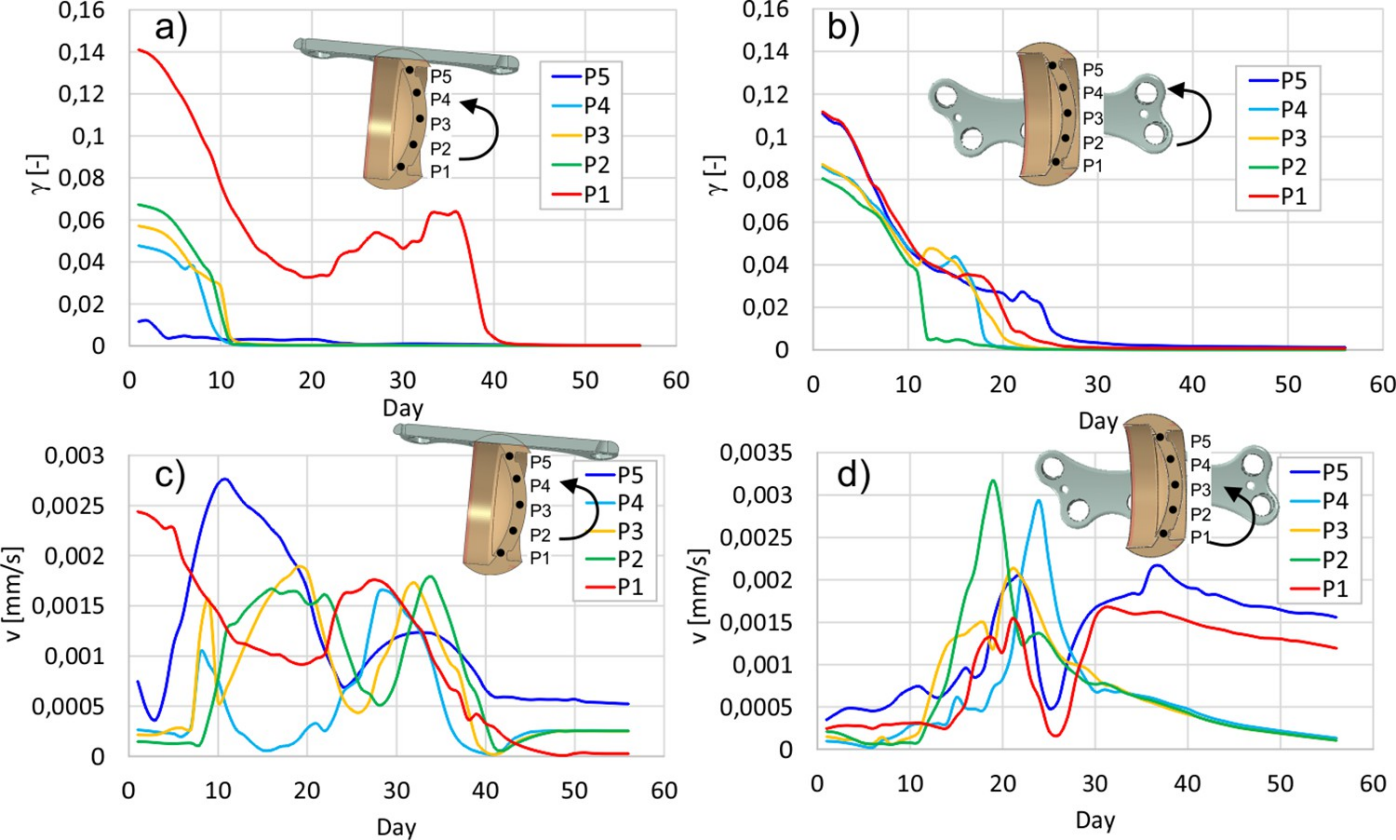
Biomechanical stimuli in selected points P1-P5 in the gap, P_max_ = 50 N; octahedral shear strain: a) dorsal plate, b) medial plate; fluid velocity: c) dorsal plate, d) medial plate.

Fig 6 illustrates the biomechanical stimuli, i.e. octahedral shear strain and interstitial fluid velocity, during the healing at five characteristic points (P1-P5) placed in the fracture gap. As reported in [21] the velocities fluctuate due to the stiffness and permeability changes. Typically, the strain decrease is followed by the velocity increase. The effect is more intensive during the bone tissues formation which are characterized by higher permeability than cartilage. The velocity increase may be explained by growth of the pore pressure gradient caused by the stiffness changes.

The further progress of stiffening inhibits the fluid flow due to the reduction of pressure gradient. Especially Fig 6A and 6B illustrate the differences in octahedral shear strain within the fracture site obtained for two fixator placements. The dorsal plate provides non-symmetrical stiffness distribution in the callus, stimulating large discrepancies of the strain between the top and bottom part of the fracture. On the other hand, the medially positioned plate guaranties the symmetrical stiffness distribution and more uniform strain field in the callus.

**Fig 7:**
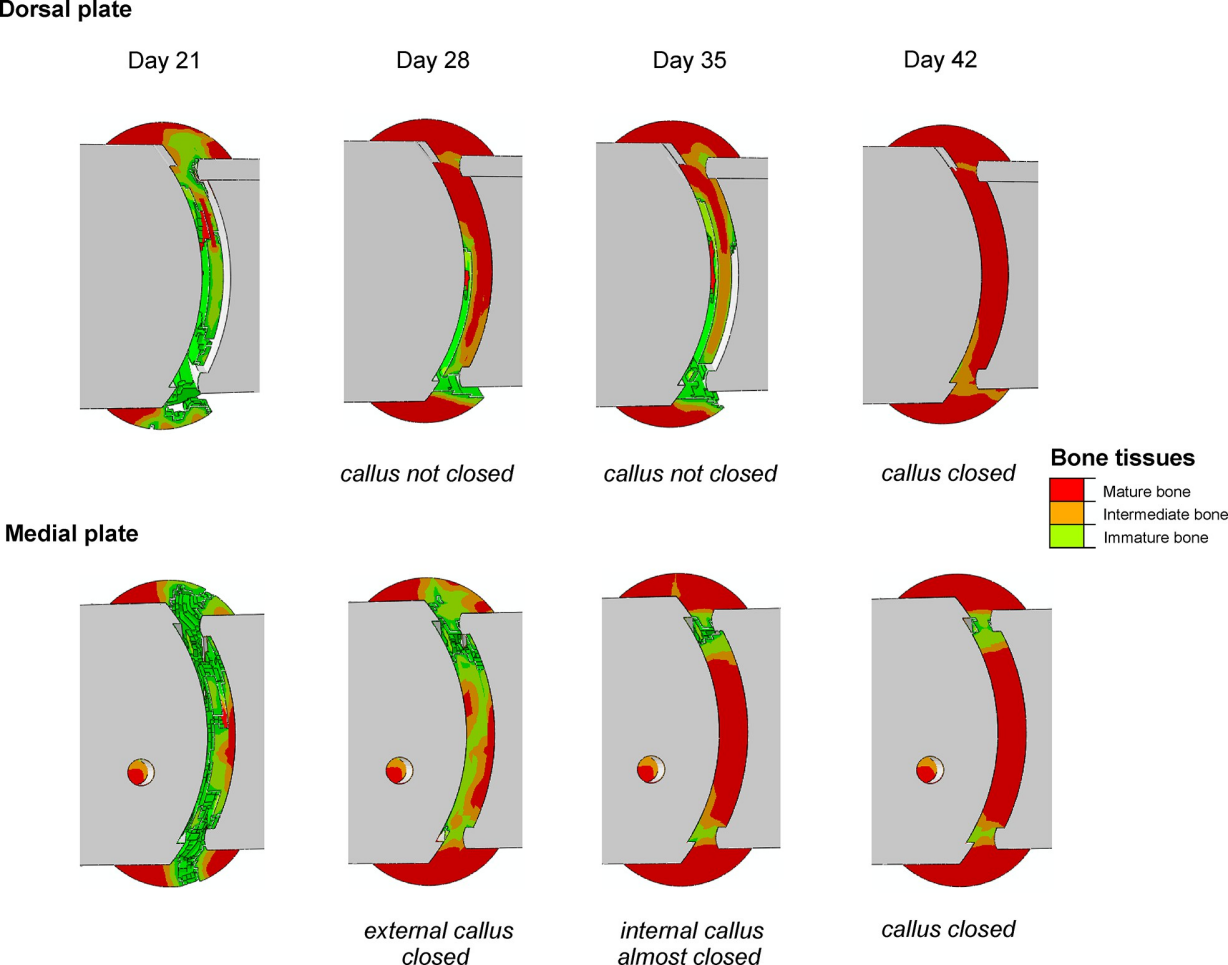
Bone bridging progress in weeks 4–6, P_max_ = 50 N.

In Fig 7 the bone bridging progress between the 4^th^ and 6^th^ week is presented. It is clear, that the medial plate promotes faster closing of the external callus and bridging of its internal part. In contrast the dorsal plate does not allow the callus closing up to the end of the 7^th^ week.

## Discussion

In the paper the comparison of local mechanical conditions in the callus tissue after first metatarsophalangeal joint arthrodesis for different placement of locking plate is presented. Dorsally positioned plate is regarded as gold standard. However, as shown by [11], the stabilisation technique with the use of medial plate seems to be more advantageous from the medical point of view, since it is less invasive. Additionally, in [12] it was shown, that medial plate approach yields lower risk of hardware failure and provides higher stiffness of the stabilized region.

The present study is made within the framework of the finite element method. The healing process is assumed to be driven by the mechanoregulation theory of tissue differentiation proposed by Prendergast. As the healing progress is qualitatively comparable in both considered loading cases (25 N and 50 N) and that the differences between the both plates are much more pronounced for the larger maximum load of 50 N, only this load case is discussed more extensively.

Similarly as in other works concerning the healing process of e.g. long bones [48, 49], we have observed desired mechanical conditions for a new bone formation close to the periosteum in the first healing stage. It is due to low biomechanical stimulus and large amount of MSC in this region. This is consistent with histological observations [18] and other FEM investigations [50]. According to Charnley [51], initial interfragmentary movements between bones may be limited by increasing the mass of external callus.

Concerning medially positioned plate it can be noticed that almost the entire gap is filled with the bone tissues at the end of the 4^th^ week (Fig 7). The external callus closes at this stage, restraining the deformations of the gap [51], and from this moment the healing progress becomes driven mainly by the fluid flow in the region between the cortical bone fragments (Fig 6B and 6D, points P1, P5). The significant pore pressure gradient appears here due to large values of pore pressure in the compression zone of external callus. The resulting increase of the fluid velocity (Fig 6D) inhibits formation of the mature bone in this region. This adverse effect is less pronounced in the tension zone due to the capillary effects which reduce the pore pressure gradients. However, after 6 weeks the bone bridging is achieved almost in entire gap except of the small area in the compression zone (Fig 7). Nonetheless, it has little medical significance.

The dorsal plate promotes formation of the hard tissues in the compression zone (Figs 4 and 7). In the initial phase, i.e. up to 10^th^ day, both the strains and fluid velocities affect the healing progress (Fig 6A and 6C). The gradual stiffening of the gap and external callus, running downward from the compression zone (Fig 4), provides the caudal translation of the bending neutral surface, leading to the decrease of the strains magnitudes in the gap (Fig 6A). The callus hardening in the thickness direction intensifies in the 4^th^ and 5^th^ weeks (Fig 7). Fluid velocity magnitudes vary due to the stiffness changes but consequently reduce since 5^th^ week. The later stage of the healing process for the dorsal plate is driven mostly by the deformation. As at ca. 40^th^ day the external callus is totally filled with the mature bone, the deformations are limited and the bone bridging is achieved after 6 weeks.

Comparison of Fig 5A and 5B reveals that during the first 5 weeks the healing progress runs generally faster for the medially positioned plate. In the ca. 17^th^ day, only bone and cartilaginous tissues were observed within the callus for the arthrodesis with medial plate. On the contrary, in the case of the dorsal plate the granulation tissue remains in some regions up to ca. 38^th^ day (Fig 5A, Fig 5C). From Figs 4, 5 and 7 it follows, that medially positioned plate enables earlier formation of hard external and medullary callus. These results are in good agreement with the findings of previous works that showed a great reduction of the healing time when the stiffness of the fixation device was increased [20, 49, 52, 53]. However, one has to notice, that too stiff fixation may inhibit the callus formation [53–55]. In fact the healing outcome is result of an interplay between the mechanical and biological processes which are crucial for, e.g., the callus formation.

One of the essential limitation of the present FEM model is the arbitrary definition of the initial shape of the callus. Some modern approaches simulate this stage of healing [48, 55]. The other limitations of the model are simplified geometry of the bones, external loads limited to ground reaction force in the toe-off phase of gait and omitting of the proliferation process. These issues are planned to be addressed in future studies.

## Conclusions

Presented results suggest, that after a certain period, 6 weeks in the simulation, the mechanical conditions for bone formation are comparable for both plates, which is consistent with clinical trials of [11]. However, the medially positioned plate provides a mechanically attractive conditions for earlier bone bridging because it maintains the symmetry of the callus, which allows for a more uniform load distribution in the fracture region and earlier hardening of the medullary callus.

## Supporting information

S1 Data (XLSX)
